# Time-sequential change in immune-related gene expression after irradiation in glioblastoma: next-generation sequencing analysis

**DOI:** 10.1080/19768354.2021.1954550

**Published:** 2021-07-30

**Authors:** Yi-Jun Kim, Kwangsoo Kim, Soo Yeon Seo, Juyeon Yu, Il Han Kim, Hak Jae Kim, Chul-Kee Park, Kye Hwa Lee, Junjeong Choi, Myung Seon Song, Jin Ho Kim

**Affiliations:** a Department of Radiation Oncology, Seoul National University College of Medicine, Seoul, Republic of Korea; b Institute of Convergence Medicine, Ewha Womans University Mokdong Hospital, Seoul, Republic of Korea; c Transdisciplinary Department of Medicine & Advanced Technology, Seoul National University Hospital, Seoul, Republic of Korea; d Cancer Research Institute, Seoul National University College of Medicine, Seoul, Republic of Korea; e Institute of Radiation Medicine, Medical Research Center, Seoul National University, Seoul, Republic of Korea; f Department of Neurosurgery, Seoul National University College of Medicine, Seoul, Republic of Korea; g Department of Information Medicine, Asan Medical Center and University of Ulsan College of Medicine, Seoul, Republic of Korea; h Yonsei Institute of Pharmaceutical Sciences, College of Pharmacy, Yonsei University, Incheon, Republic of Korea; i Department of Psychiatry, Keyo Hospital, Uiwang, Republic of Korea

**Keywords:** Glioblastoma, Radiotherapy, Immune-related signals, Next-generation sequencing, Transcriptome

## Abstract

The time-sequential change in immune-related gene expression of the glioblastoma cell line after irradiation was evaluated to speculate the effect of combined immunotherapy with radiotherapy. The U373 MG glioblastoma cell line was irradiated with 6 Gy single dose. Next-generation sequencing (NGS) transcriptome data was generated before irradiation (control), and at 6, 24, and 48 h post-irradiation. Immune-related pathways were analyzed at each time period. The same analyses were also performed for A549 lung cancer and U87 MG glioblastoma cell lines. Western blotting confirmed the programmed death-ligand 1 (PD-L1) expression levels over time. In the U373 MG cell line, neutrophil-mediated immunity, type I interferon signaling, antigen cross-presentation to T cell, and interferon-*γ* signals began to increase significantly at 24 h and were upregulated until 48 h after irradiation. The results were similar to those of the A549 and U87 MG cell lines. Without T cell infiltration, PD-L1 did not increase even with upregulated interferon-*γ* signaling in cancer cells. In conclusions, in the glioblastoma cell line, immune-related signals were significantly upregulated at 24 and 48 h after irradiation. Therefore, the time interval between daily radiotherapy might not be enough to expect full immune responses by combined immune checkpoint inhibitors and newly infiltrating immune cells after irradiation.

## Background

Recently, both phase III studies of nivolumab, an immune checkpoint inhibitor of programmed death-1 (PD-1) plus radiotherapy in newly diagnosed glioblastoma (CheckMate-548 (NCT02667587) and -498(NCT02617589)) failed to meet the primary endpoint (Weller and Le Rhun 2019). In these trials, patients received nivolumab every two weeks in addition to radiotherapy, and then every four weeks. On the other hand, recent studies reported that neoadjuvant nivolumab two weeks before surgery in recurrent glioblastoma was successful, although the sample size was small (Cloughesy et al. 2019). Therefore, well-designed clinical trials of immunotherapy may improve survival even in newly diagnosed glioblastoma.

Radiotherapy in glioblastoma is an indispensable treatment. The gold standard of treatment for newly diagnosed glioblastoma is gross total resection followed by standard brain radiotherapy with concurrent and adjuvant temozolomide. The recommended radiation dose is 60 Gy in 30 fractions of 2.0 Gy 5 days per week (Stupp et al. 2005). Immunotherapy has been a promising strategy after successful trials in several cancer types. Among them, immune checkpoint inhibitors targeting PD-1/programmed death ligand-1 (PD-L1) and cytotoxic T-lymphocyte-associated protein 4 (CTLA-4) has been widely tested and approved as treatment options for solid organ cancers such as non-small cell lung cancer, renal cell carcinoma, and melanoma (Wolchok et al. 2013; Motzer et al. 2015; Antonia et al. 2017). Since glioblastoma is one of the most aggressive and incurable diseases (Tulip et al. 2021), immunotherapy studies for glioblastoma have been extensively conducted. Until now, however, no immunotherapy has been approved by the FDA for glioblastoma (McGranahan et al. 2019). Unlike radiotherapy, immunotherapy for glioblastoma is still in the investigation phase.

Radiation generates tumor antigens by killing tumor cells (Wang et al. 2018). After innate immune activation by neutrophils, antigen-presenting cells absorb tumor-derived DNA to activate the STING pathway. This pathway produces type I interferons, resulting in the recruitment and activation of dendritic cells (Burnette et al. 2011). Cross-presentation of tumor antigen to naïve CD8+ T cells is carried out by the dendritic cells. In addition, radiation-induced interferon-*γ* upregulates the class I major-histocompatibility-complex (MHC) expression, allowing T cells to recognize tumor cell targets and infiltrate into tumors (Lugade et al. 2008; Shao et al. 2017). Interferon-*γ* is also secreted from these recruited T cells to inhibit the tumor cell cycle. If the tumor is not eliminated, cancer cells upregulate PD-L1 by interferon-*γ* from T cells and the infiltrating T cells become dysfunctional (Trujillo et al. 2018). Anti-PD-(L)1 therapy reactivates these infiltrating T cells to kill cancer cells (Deng et al. 2014). Therefore, radiotherapy eventually increases tumor antigen, type I interferon, interferon-*γ*, and PD-L1 in tumor cells.

Radiation to the tumor target field directly kills not only tumor cells but also infiltrating immune cells (Schaue and McBride 2012). Therefore, it may take time to recruit new immune cells to the tumor tissue by radiation-induced signaling. It is not well studied how long time will be needed for the optimal effects on immunotherapy by infiltrating immune cells.

Also, several studies reported that the loss of interferon-*γ* and antigen-presenting pathway genes in cancer cells causes resistance to PD-1 and anti-CTLA-4 therapies (Gao et al. 2016; Zaretsky et al. 2016). It suggests that not only infiltration of immune cells into the tumor microenvironment, but the intact related pathways in tumor cells is also an indispensable factor for immunotherapy. Therefore, measuring the time to upregulate immune-related signals in cancer cells can provide clues for the optimal time point of immunotherapy combined with radiotherapy.

In this study, we observed how immune-related signaling changes after irradiation in the glioblastoma cell line overtime to find clues of the most effective methods in the combination between immunotherapy and radiotherapy.

## Methods

### Cell culture and irradiation

The human glioblastoma cell line, U373 MG (Uppsala), with inactive mutant p53, was used in this study (Korean cell line bank [KCLB] no. 30017, http://cellbank.snu.ac.kr/english/) (Kim et al. 2004). Cells were cultured in RPMI1640 media (Welgene), supplemented with 10% fetal bovine serum and 1% gentamicin, at 37°C in water saturated with 5% CO_2_. Referring previous studies (Hyun et al. 2011; Ma et al. 2013; Kang et al. 2019), ionizing irradiation dose of 6 Gy was selected and delivered to the cell line (cell number, 5 × 10^5^/100 mm dish) using 6 MV X-ray at 400 MU/min rate via a linear accelerator (Clinac 2100 C or Clinac 21EX, Varian Medical Systems). For comparison with a wild type cell line, a cell line of human lung cancer, A549, was also cultured under the same conditions (KCLB no.10185) (Kim et al. 2004; Kim et al. 2013). Authentication and mycoplasma tests were performed on both U373 MG and A549 cell lines (CosmoGenetech, Seoul, Korea) (Supplementary Figure S1).

We extracted open source data of human U87 MG glioblastoma cell line (ATCC, Bethesda, MD) from the gene expression omnibus (GEO, accession no.: GSE56937). The original study demonstrates cell culture and irradiation protocols for this cell line (McDonald et al. 2017). Briefly, the U87 MG cell line was cultured in minimum essential medium (MEM) with 10% fetal bovine serum, maintained in a 37°C incubator with 5% CO_2_. Cultures were treated with single doses of irradiation (8 and 16 Gy) via Cesium-137 Gammacell irradiator with a dose-rate of 0.48 Gy/min. We chose data with 8 Gy irradiation, which is similar to our study setting of 6 Gy.

### Ribonucleic acid (RNA) isolation, library construction and sequencing

RNA isolation and Next-generation sequencing (NGS) were performed on the U373 MG and A549 cell lines before irradiation (0 h, control group), and 6, 24, and 48 h (test groups) post-irradiation. The total RNA concentration was calculated using the Quant-IT RiboGreen (Invitrogen). Samples were run on TapeStation RNA ScreenTape (Agilent) to assess the integrity of the total RNA. Only high-quality RNA preparations (RNA integrity number > 7.0) were used for RNA library construction.

A library was prepared using 1 μg of total RNA from each sample using the Illumina TruSeq mRNA Sample Prep kit (Illumina, Inc.). First, the poly (A)-containing mRNA molecules were purified using poly-T-attached magnetic beads. Then, the mRNA was fragmented into small pieces with divalent cations at elevated temperatures. SuperScript II reverse transcriptase (Invitrogen) and random primers were used to copy the cleaved RNA fragments into the first-strand cDNA. Subsequently, second-strand cDNA synthesis using DNA polymerase I and RNase H were performed. These cDNA fragments then underwent an end repair process, the addition of a single ‘A’ base, and the ligation of indexing adapters. The products were purified and enriched via PCR to generate the final cDNA library. The libraries were quantified using qPCR, according to the qPCR Quantification Protocol Guide (KAPA Library Quantification Kits for Illumina sequencing platforms), and validated using TapeStation D1000 ScreenTape (Agilent Technologies). Indexed libraries were then sequenced on the HiSeq2500 platform (Illumina) by Macrogen Incorporated.

The study that provided the corresponding data for the U87 MG cell line harvested cells at various time points postirradiation. We chose time points similar to our study (1, 4, 6 days after irradiation).

### Bioinformatics and statistical analysis

The fragments per kilobase of transcript per million mapped reads (FPKM) of genes for each period (0, 6, 24, and 48 h) were extracted, and genes with more than five FPKM in at least one period alone were selected for further analysis of the U373 MG and A549 cell lines. For these genes, the log2-fold change (LogFC), comparing the expression level between the test groups (6, 24, 48 h, post-irradiation) and the control group (0 h, before irradiation), was calculated.

First, a heatmap was generated according to the LogFC value in each period using the Morpheus program (https://software.broadinstitute.org/morpheus/). After hierarchical analysis according to the LogFC patterns over time, functional enrichment analysis for each clustered group was performed using the Enrichr (https://amp.pharm.mssm.edu/Enrichr/enrich) and Reactome programs (https://reactome.org/).

Second, in each time group (6-, 24-, and 48-hour groups), genes upregulated than before irradiation with a fold change ≥1.2 (LogFC ≥0.263034) were extracted. Using the Enrichr and Reactome programs, functional enrichment analyses for the gene sets were performed based on the gene ontology (GO) terms and the Reactome’s stable identifiers, respectively. We mainly observed the time-sequential change of immune-related signals including the expression level of neutrophil-mediated immunity, type I interferon, antigen-presenting, and interferon-*γ* signals in tumor cells. The false discovery rate (FDR)-based adjusted *P*-value (FDR *Q*-value) was automatically evaluated. A *P*- or *Q*-value < 0.05 was considered statistically significant.

Third, we examined the time-sequential change in LogFC values of PDL1 and TGFB1 which encode PD-L1 and TGF-ß1 in the NGS data.

### Comparison with p53 wild type glioblastoma cell lines

Cell culture, irradiation, RNA isolation, sequencing, and functional enrichment analysis were performed for the U87 MG cell line using the same methods as for the U373 MG cell line. In the GEO datasets, the p53 wild type glioblastoma cell line (U87 MG) dataset was selected (GSE56937). Six samples obtained, three each from the control (no irradiation) and test (8 Gy irradiation) groups after day one were compared because the test scheme was similar to our study. By using the GEO2R program, LogFC was automatically calculated. Genes with a fold change ≥1.2 (LogFC ≥0.26) were extracted and functional enrichment analysis was performed. The result was compared with that of the U373 MG and A549 cell lines.

### Western blotting

Cell lysates were prepared in cell lysis buffer (iNtRON Biotechnology). The total cellular proteins were separated via SDS-PAGE (10 μg for each period) and transferred to nitrocellulose membranes (Millipore Corp.). The membranes were blocked with blocking solution in 5% nonfat dry milk (25 mM Tris, pH 7.5; 0.15 M NaCl; 0.05% Tween) for 1 h and probed overnight at 4°C with primary rabbit polyclonal IgG antibody at a dilution of 1:1000 (in Double blocker, T&I BDT-1000). The antibodies for CD274 and TGFB1 were purchased from Cell Signaling Technology (Beverly). The membranes were incubated with blocking solution containing a dilution of HRP-conjugated goat anti-rabbit IgG as a secondary antibody (Santa Cruz, Biotechnology) at 1:3000 for 2 h. Western blot protein detection was performed using the ECL kit (Intron Biotechnology) according to the manufacturers’ recommendations. As a control, a monoclonal antibody against actin (Santa Cruz, Biotechnology) was used. The expression level of PDL1 and TGFB1 of the U373 MG cell line were examined via western blotting. The cells were treated with media, irradiated with 6 Gy, and then collected at 0 h (before irradiation), and then, 6, 24, and 48 h after radiation, as for the previous NGS data analysis.

## Results

### Each immune pathway expression changed differently overtime after irradiation

We examined the time-sequential change of immune-related pathways after irradiation in glioblastoma to estimate the effect of combined immuno-radiotherapy. We hypothesized that the time-series characteristics of regulation after radiation may differ for each immune-related pathway. Since conventional radiotherapy for glioblastoma is a daily schedule, we examined the time-sequential change pattern of radiation-induced expression up to 6, 24, and 48 h after irradiation.

After NGS of the U373 MG cell line at each designated time period post-radiation, genes with >5 FPKM in at least one period were selected. A total of 9278 genes were detected. A hierarchical heatmap analysis of LogFC value of each gene at each time period generated four clusters with the gene numbers 2572, 631, 5503, and 572.

**Figure 1.**
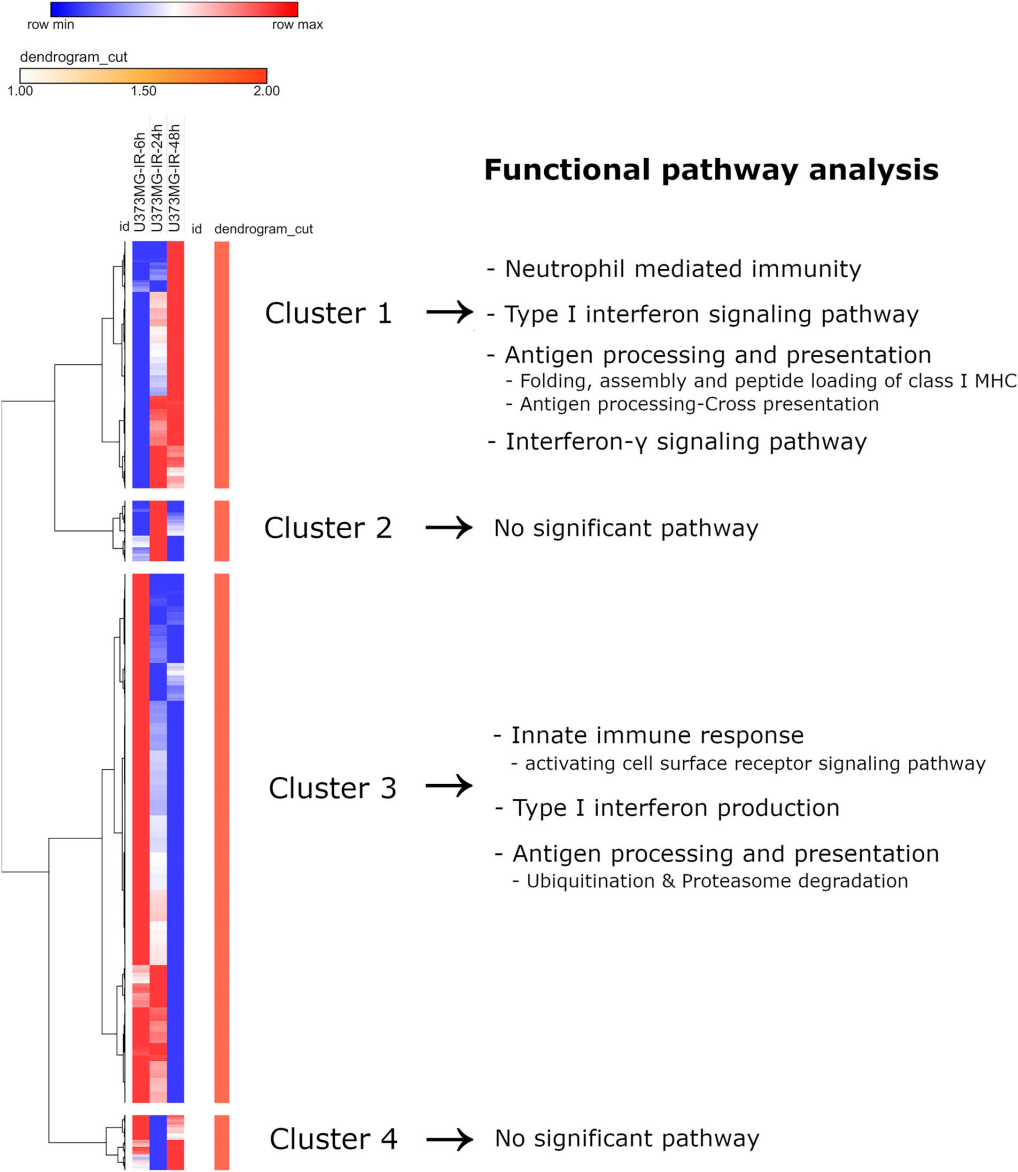
Heatmap of the log2-fold change for each gene at 6, 24, and 48 h post-irradiation compared to that at 0 h (no-irradiation) and functional pathway analysis for each cluster group in the U373 MG glioblastoma cell line showed that the highly expressed time points after irradiation are different for each immune pathway. IR-6h, 6 h after irradiation; IR-24h, 24 h after irradiation; IR-48h, 48 h after irradiation.

Functional enrichment analysis was performed for each of these four gene groups (Figure 1). The first gene group (cluster 1) showed a gradual rise in gene expression. Gene expression in the second gene cluster group (cluster 2) increased in 24 h and decreased in 48 h post-irradiation. Gene expression in the third group (cluster 3) decreased over time, while expression in the fourth group increased 48 h post-irradiation (cluster 4).

Gene expressions of innate immune response activating cell surface receptor signaling pathway increased at 6 h after irradiation and decreased over time (cluster 3; GO:0002220; *P* = 1.63E-08; FDR *Q* = 4.27E-07; Odds ratio [OR], 1.861), while the neutrophil-mediated immunity increased gradually until 48 h (cluster 1; GO:0002446; *P* = 7.50E-13; FDR *Q* = 3.83E-09; OR, 1.912).

Type I interferon was actively produced at 6 h after irradiation (cluster 3; GO:0032481; *P* = 2.55E-05; FDR *Q* = 3.66E-04; OR, 1.904) and the cellular response to type I interferon was gradually upregulated until 48 h (cluster 3; GO:0071357; *P* = 2.44E-07; FDR *Q* = 7.79E-05; OR, 2.945).

The expression of antigen processing and presentation related to ubiquitination and proteasome degradation was predominant at 6 h after irradiation and reduced over time (cluster 3; R-HSA-983168; *P* = 1.32E-11; FDR *Q* = 5.12E-09), while the expression levels of folding, assembly and peptide loading of class I MHC molecules (cluster 1; R-HSA-983170; *P* = 5.67E-10; FDR *Q* = 4.60E-07) and cross-presentation to stimulate CD8+ T cell immunity (cluster 1; R-HSA-1236975; *P* = 4.66E-05; FDR *Q* = 0.014) were progressively upregulated until 48 h after irradiation.

The interferon-*γ* signaling pathway was gradually upregulated over time (cluster 1; GO:0060333; *P* = 1.57E-05; FDR *Q* = 0.002108; OR, 2.519) when observed up to 48 h after irradiation.

These results suggest that immune cells (neutrophil and antigen-induced cytotoxic T cells) would be recruited toward tumor cells at least after 24 h post-irradiation, resulting in a narrow window period for immune-mediated tumor cell death before the next radiotherapy schedule in glioblastoma.

### Upregulation of immune cell activation-related pathway occurred after 24 hours post-irradiation

The previous clustering analysis was an analysis of time-sequential changes in each immune pathway. Inversely, we further assessed the significantly upregulated immune pathways in each period and verified whether the results are similar to the previous clustering analysis.

**Figure 2.**
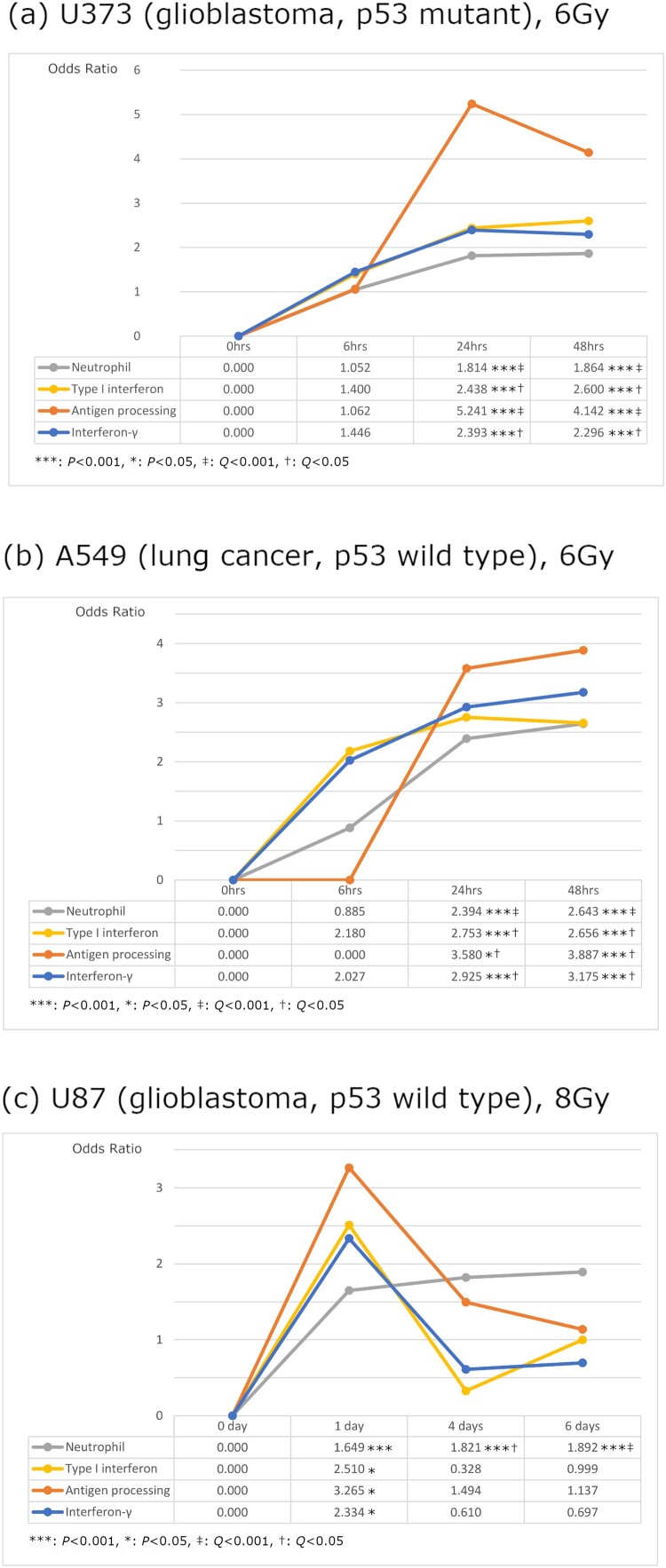
Immune-related enrichment analysis of each time period post-irradiation showed that neutrophil, type I interferon, and antigen processing, and interferon-*γ* signals are upregulated after 24 h post-irradiation. *P*: Fisher’s exact test. *Q*: Adjusted *P*-value using the Benjamini-Hochberg method.

Functional analysis for significantly upregulated genes compared to pre-irradiation with fold change >1.2 at each period (6, 24, and 48 h) in the U373 MG cell line was performed. Immune-related signals – neutrophil-mediated immunity (GO:0002446), type I interferon signaling pathway (GO:0060337), antigen processing and presentation of peptide antigen via MHC class I (GO:0002474), and interferon-*γ*-mediated signaling pathway (GO:0060333) were observed. These all signal pathways were significantly upregulated at 24 h after irradiation and the significances were maintained until 48 h (Figure 2(a)). Therefore, both time-series analysis in each immune pathway and immune pathway analysis in each time-series suggest that it would take at least 24 h for radiation-induced immune cell activation.

To compare the differential gene expression based on the p53 mutation and cell type, the same analysis was conducted with the p53 wild type of the lung cancer cell line (A549). Lung cancer cell line was selected considering a recent successful immunotherapy trial in lung cancer to analyze the difference in immunologic pathway after irradiation between glioblastoma and lung cancer (Antonia et al. 2017). Unlike the U373 MG cell line, p53-mediated apoptosis after irradiation was one of the critical mechanisms of tumor cell death at 6 h after irradiation (apoptotic process; GO: 0006915; *P* = 1.97E-08; FDR *Q* = 2.01E-05; OR, 4.341 and TP53 Regulates Transcription of Cell Death Genes; R-HSA-5633008; *P* = 1.43E-05; FDR *Q* = 0.012). However, the immune-related signals showed a similar result with the U373 MG cell line. All immune-related signals were significantly upregulated at 24 and 48 h after irradiation (Figure 2(b)).

We searched for additional datasets reporting a time-sequential change of mRNA expression after irradiation in glioblastoma cell line from the GEO and found the U87 MG cell line datasets (glioblastoma, p53 wild type, ATCC, Bethesda, MD) from a study performed at Center of Cancer Systems Biology (McDonald et al. 2017). The original study collected samples at 1, 4, 6, and 35 days after 8 Gy or 16 Gy irradiations. Among all mRNA expression data, 1, 4, and 6 days of data after 8 Gy irradiation similar to our study design were selected to observe changes in immune-related signals after irradiation in the U87 MG cell line. As results, neutrophil-mediated immunity was significantly upregulated at one day and the upregulation was maintained until six days after irradiation. While the expression levels of other signals significantly increased at one day and decreased at 4 and 6 days after irradiation (Figure 2(c)). This result shows that radiation-induced immune cell activation takes considerable time regardless of p53 mutation and tumor cell type.

The number of genes upregulated in the U373 and A549 cell lines increased over time (159, 393, and 604 in the U373 cell line and 417, 1541, and 1597 in the A549 cell line at 6, 24, and 48 h after irradiation, respectively). The number of genes commonly upregulated in both cell lines also increased (4, 89, and 176 at 6, 24, and 48 h after irradiation, respectively). This corresponds to 2.5%, 22.6%, and 29.1% of the upregulated genes in the U373 cell line. There were no statistically significant immune signals at 6 and 24 h in the enrichment analysis of these genes. However, at 48 h, the type I interferon signaling pathway was detected as a significantly increased signal (GO:0060337; *P* < 0.001; adjusted FDR *Q* = 0.026; related genes were STAT2, IFI6, ISG15, HLA-A, and IRF9). Neutrophil-mediated immunity showed borderline significance (GO:0002446; *P* = 0.001; adjusted FDR *Q* = 0.059; related genes were LGALS3, ARSA, SERPINA3, GRN, GSN, JUP, GAA, PSAP, CAT, CTSH, CTSD, and DNASE1L1). These results show that the immunologic signals increased gradually in both cell lines up to at least 48 h post-irradiation, and 24 h post-irradiation may not be the optimal time for a complete immune response.

The number of upregulated genes in the U87 cell line gradually increased to 923, 1065 and 1415 at 1, 4 and 6 days after irradiation, respectively. Genes upregulated on day 1 of the U87 cell line were compared with those of the other two cell lines 24 h after irradiation. The number of genes commonly upregulated between the U87 and U373 cell lines was 68 (7.3%, 68/923) and 250 (27.1%, 250/923) between the U87 and A549 cell lines. No significant immune signals were observed in enrichment analyses for these common genes. Gene lists are provided in Supplementary Tables S1–S5.

### PD-L1 expression level did not increase after irradiation with cancer cell alone

**Figure 3.**
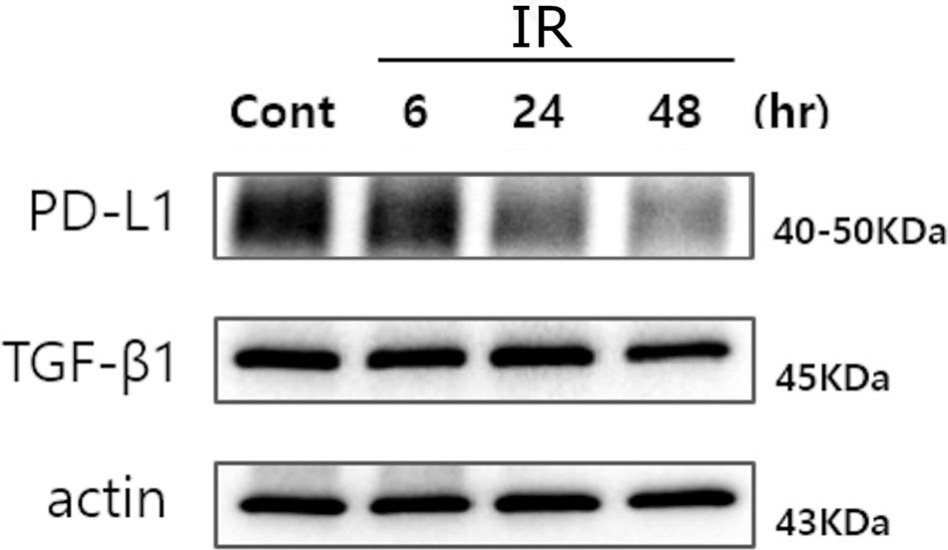
Western blotting for each time period post-irradiation of the U373 MG glioblastoma cell line showed that PD-L1 and TGF-β was not activated after irradiation until 48 h post-irradiation. IR, irradiation; Cont, control.

Although the expression level of interferon-*γ* signaling increased in the U373 MG cell line in the NGS data analysis, the expression level of PDL1 gene did not increase over time, suggesting that without T cell infiltration, the 6 Gy single dose irradiation to the U373 MG glioblastoma cell line did not upregulate PD-L1 (Table 1). Western blotting confirmed no increase of PD-L1 after irradiation in the U373 MG cell line (Figure 3) (all results of western blotting can be accessed from Supplementary Figures S2 and S3). In the A549 cell line, PDL1 gene expression level was relatively lower at all time periods compared to that of the U373 MG cell line, without markedly increasing after irradiation (Table 2).

**Table 1.** Time sequential gene expression level encoding the AXL-PI3K-PD-L1 axis and TGF-β1 at 6, 24, and 48 h after irradiation in U373 MG glioblastoma cell line.

Gene	mRNA expression level (FPKM)				Log2 fold change^a^		
	CONT-0 h	IR-6 h	IR-24 h	IR-48 h	IR-6 h	IR-24 h	IR-48 h
PDL1	16.2	9.3	3.6	5.0	−0.740	−1.898	−1.530
TGFB1	36.2	36.2	35.6	39.4	−0.0001	−0.026	0.117

FPKM, the Fragments Per Kilobase of transcript per Million mapped reads; CONT-0 h, control group with no radiation; IR, irradiation.

^a^ Comparison with the control group.

**Table 2.** Time sequential gene expression level encoding the AXL-PI3K-PD-L1 axis and TGF-β1 at 6, 24, and 48 h after irradiation in A549 lung cancer cell line.

Gene	mRNA expression level (FPKM)				Log2 fold change^a^		
	CONT-0 h	IR-6 h	IR-24 h	IR-48 h	IR-6 h	IR-24 h	IR-48 h
PDL1	0.3	0.1	0.3	0.3	−0.139	−0.002	0.063
TGFB1	43.4	38.3	50.5	62.9	−0.176	0.213	0.524

FPKM, the Fragments Per Kilobase of transcript per Million mapped reads; CONT-0 h, control group with no radiation; IR, irradiation.

^a^ Comparison with the control group.

These results indicate that radiation-induced PD-L1 expression was not upregulated within 24 h of the radiotherapy interval with tumor cells alone. Therefore, if there is no increase in T cell-mediated PD-L1 expression in tumor cells, it is difficult to expect synergistic effects in concurrent immune-radiotherapy.

### The TGF-β1 did not upregulated after irradiation

One study suggested that a combination of anti-PD-L1 and anti-TGF-ß1 therapies may overcome immunotherapy resistance, as TGF-β forms a tumor microenvironment to inhibit anti-tumor immunity by restricting T-cell infiltration (Mariathasan et al. 2018). Therefore, we observed the time-sequential change in LogFC values of TGFB1 which encode TGF-ß1.

The TGF-β1 level was relatively stable with logFC values of −0.0001, −0.026, and 0.117 at 6, 24, and 48 h post-irradiation, respectively (Table 1). Western blotting was performed for TGF-β1 to confirm the stable expression regardless of the period, suggesting that irradiation of 6Gy single dose did not impact the TGF-β1 expression in the U373 MG cell line (Figure 3). Even in the A549 cell line, increased expression of the TGFB1 expression was stable irrespective of radiation (Table 2). This result suggests that anti-TGF-β might be used irrespective of radiotherapy schedule.

## Discussion

Our study observed the time-sequential change in immune-related gene expressions after irradiation of glioblastoma cells. We mainly focused on speculating the optimal time points for combining radiotherapy and anti-PD-(L)1 treatment. We found that neutrophil-mediated immunity, antigen processing/cross-presentation to activate cytotoxic T cells, and radiation-induced-interferon-*γ*-related signals in tumor cells were highly upregulated at 24 h after irradiation and maintained until 48 h. It suggests that tumor antigen-specific T cells would infiltrate to tumor cells after 24 h from radiation, and the PD-L1 increase in tumor cells by interferon-*γ* from the infiltrating T cell would take more than 24 h after irradiation. Therefore, concurrent anti-PD-(L)1 therapy with daily fractionated radiotherapy might not be fully effective in glioblastoma because of the time required for re-infiltration of tumor antigen-specific T cells after the previous infiltrating T cells were killed by radiotherapy.

Radiation increases PD-L1 in tumor cells. Radiation increases interferon-*γ* by infiltrating T cell, and increased interferon-*γ* provokes tumor cells to generate PD-L1 to make T cells dysfunctional (Lugade et al. 2008; Gerber et al. 2013; Abiko et al. 2015). T cell-mediated-interferon-*γ* is thought to be the main mechanism behind increasing PD-L1 levels (Abiko et al. 2015). Also, radiation damages tumor cells, and as damage responses, cancer cells itself express PD-L1 without T cells via the base repair system (Permata et al. 2019), and the DNA double-strand break repair pathway (Sato et al. 2017). Several studies, using a tumor cell line only, have reported increased PD-L1 expression post-irradiation (Sato et al. 2017; Permata et al. 2019).

In our study, although interferon-*γ* signaling increased after irradiation, PD-L1 expression did not increase. There are two possible causes. First, we observed immune signal regulations only up to 48 h after irradiation, which may have been a short time to detect an increase in PD-L1 by radiation-induced interferon-*γ*. Several in vitro and in vivo studies reported that interferon-*γ* increases approximately after 2 days (1-6 days) after irradiation (Gerber et al. 2013; Lim et al. 2014; Sato et al. 2017; Permata et al. 2019). Interferon-*γ* may be produced by tumor cells after irradiation or by T cells recruited after antigen-presenting process. Interferon-*γ* generated by tumor cells recruits T cells, and interferon-*γ* produced by T cells increases PD-L1 in tumor cells. In our study, increased interferon-*γ* signaling was derived from tumor cells (not T cells). Therefore, the results of this study suggest that T cell infiltration induced by interferon-*γ* from tumor cells rarely occurs until at least 1–2 days after irradiation.

Second, since there was no T cell in our cell line study, the amount of PD-L1 might be less than when T cells are present. When T cells were inhibited, the amount of interferon-*γ* was reduced significantly (Gerber et al. 2013). Autocrine signaling by interferon-*γ* produced in tumor cells may increase PD-L1, but the release of interferon-*γ* by T cells is much more massive and is thought to play a pivotal role in PD-L1 expression. This result suggests that without T cell infiltration and interferon-*γ* secreted by T cells, anti-PD-(L)1 therapy may not be effective.

Recent trials showed improved results when radiotherapy and immunotherapy were combined as an adjuvant (after radiotherapy) or neoadjuvant (before radiation) settings. The Pacific trial demonstrated a survival benefit from the adjuvant anti-PD-1 therapy after chemo-radiotherapy in stage III non-small-cell lung cancer (Antonia et al. 2017). Without surgery, chemo-radiotherapy would upregulate tumor antigen process and presentation and PD-L1 level in tumor cells would also be highly expressed after completing chemo-radiotherapy. This study demonstrated that patients with time interval between chemo-radiotherapy and immunotherapy less than 14 days showed favorable survival compared to those with time interval 14 days or more.

According to our study, significant upregulation of adaptive immunity-related tumor antigen presentation started at 24 h after irradiation, suggesting that 14 days was enough time for infiltrating T cells in tumor cells. Also, as the chemo-radiotherapy-induced immune responses might be reduced over time, resulting in lower survival benefits for patients from immunotherapy.

A recent trials of neoadjuvant anti-PD-1 therapy before surgery in recurrent glioblastoma also showed a promising result (Cloughesy et al. 2019). In this study, anti-PD-1 was administered 14 days before surgery. Intratumoral T cells would be sufficiently activated by anti-PD-1 therapy before surgery. Based on this success, it would be also possible to try neoadjuvant immunotherapy for newly diagnosed glioblastoma.

On the other hand, recent two phase III trials which failed to meet the primary endpoint were concurrent anti-PD-1 therapy with (chemo)-radiotherapy in newly diagnosed glioblastoma after surgery (NCT02667587, NCT02617589) (Weller and Le Rhun 2019). Without gross tumor tissue, the PD-L1 expression level might not be prominent than the Pacific trial. Moreover, daily radiotherapy may not provide sufficient time for new T cell infiltration to tumor cells.

Concurrent immuno-radiotherapy has another point to weaken the effectiveness of the combined therapy. Anti-PD-L1 therapy activates infiltrating T cells which are dysfunctional because of PD-L1 from tumor cells. Therefore, to make a success of anti-PD-(L)1 treatment, enough density of infiltrating tumor antigen-specific T cells is indispensable. Radiotherapy affects all cells in the target field. Moreover, lymphocytes are radio-sensitive. Therefore, after irradiation, it would require time to re-infiltrate new T cells in the tumor microenvironment.

PD-L1 expression was higher in the p53 mutant tumor than in the p53 wild type cancer, and the p53 mutant tumor was highly sensitive to the anti-PD-L1 therapy (Dong et al. 2017). As the apoptotic process is not available in the p53 mutant cancer, anti-PD-(L)1 treatment might be a promising option for this disease (Aubrey et al. 2018).

Generally, radiation-induced TGF-ß1 signaling is associated with normal tissue injury and fibrosis (Vujaskovic 2000). TGF-ß signaling is now regarded as one of the possible mechanisms of immunotherapy resistance (Wrzesinski et al. 2007; Bouquet et al. 2011; Vanpouille-Box et al. 2015; Darragh et al. 2018; Mariathasan et al. 2018). Co-administration of anti-TGF-β1 and anti-PD-(L)1 (±radiotherapy) extended survival in a mouse model (Vanpouille-Box et al. 2015; Mariathasan et al. 2018). In our study, 6 Gy single dose did not increase TGF-ß1 expression. Radiation protocol without TGF-ß1 increase and/or anti-TGF-β1 therapy might be a new strategy to improve survival in glioblastoma.

In our study, we used various tumor cell lines. Further studies using tumor tissues such as heterogeneous tumor spheres or organoids or various preclinical and clinical studies would be helpful to deepen the understanding of the time-sequential change of immune response induced by radiotherapy. Administration of temozolomide, anti-PD-L1 treatment, and various doses/fractions of radiotherapies were not performed. As a time-sequential NGS study, however, this study provides a comprehensive and in-depth understanding of gene expression post-irradiation and offers a valuable recommendation for the optimal treatment protocol for combined radiotherapy and immunotherapy.

In conclusion, this study observed a time-sequential change in gene expression of glioblastoma cell line post-irradiation. Innate and adaptive immune signals were significantly upregulated at 24 h post-irradiation and maintained until 48 h. These results suggest that daily radiotherapy might not provide sufficient time for infiltrating immune cells into the tumor microenvironment. Therefore, anti-PD-(L)1 therapy against T cell-mediated PD-L1 in tumor cells might not be fully synergistic with daily radiotherapy treatment schedule.

## Supplementary Material

Supplemental Material

## Data Availability

Sequencing data were deposited in NCBI’s Sequence Read Archive (SRA) and are accessible through BioProject accession number PRJNA642705.

## References

[CIT0001] Abiko K, Matsumura N, Hamanishi J, Horikawa N, Murakami R, Yamaguchi K, Yoshioka Y, Baba T, Konishi I, Mandai M. 2015. IFN-γ from lymphocytes induces PD-L1 expression and promotes progression of ovarian cancer. Br J Cancer. 112:1501. 25867264 10.1038/bjc.2015.101 PMC4453666

[CIT0002] Antonia SJ, Villegas A, Daniel D, Vicente D, Murakami S, Hui R, Yokoi T, Chiappori A, Lee KH, de Wit M. 2017. Durvalumab after chemoradiotherapy in stage III non-small-cell lung cancer. N Engl J Med. 377:1919–1929. 28885881 10.1056/NEJMoa1709937

[CIT0003] Aubrey BJ, Kelly GL, Janic A, Herold MJ, Strasser A. 2018. How does p53 induce apoptosis and how does this relate to p53-mediated tumour suppression? Cell Death Differ. 25:104. 29149101 10.1038/cdd.2017.169 PMC5729529

[CIT0004] Bouquet F, Pal A, Pilones KA, Demaria S, Hann B, Akhurst RJ, Babb JS, Lonning SM, DeWyngaert JK, Formenti SC. 2011. TGFβ1 inhibition increases the radiosensitivity of breast cancer cells in vitro and promotes tumor control by radiation in vivo. Clin Cancer Res. 17:6754–6765. 22028490 10.1158/1078-0432.CCR-11-0544 PMC3724539

[CIT0005] Burnette BC, Liang H, Lee Y, Chlewicki L, Khodarev NN, Weichselbaum RR, Fu Y-X, Auh SL. 2011. The efficacy of radiotherapy relies upon induction of type I interferon–dependent innate and adaptive immunity. Cancer Res. 71:2488–2496. 21300764 10.1158/0008-5472.CAN-10-2820 PMC3070872

[CIT0006] Cloughesy TF, Mochizuki AY, Orpilla JR, Hugo W, Lee AH, Davidson TB, Wang AC, Ellingson BM, Rytlewski JA, Sanders CM. 2019. Neoadjuvant anti-PD-1 immunotherapy promotes a survival benefit with intratumoral and systemic immune responses in recurrent glioblastoma. Nat Med. 25:477. 30742122 10.1038/s41591-018-0337-7 PMC6408961

[CIT0007] Darragh LB, Oweida AJ, Karam SD. 2018. Overcoming resistance to combination radiation-immunotherapy: a focus on contributing pathways within the tumor microenvironment. Front Immunol. 9:3154. Epub 2019/02/16. 30766539 10.3389/fimmu.2018.03154 PMC6366147

[CIT0008] Deng L, Liang H, Burnette B, Beckett M, Darga T, Weichselbaum RR, Fu Y-X. 2014. Irradiation and anti-PD-L1 treatment synergistically promote antitumor immunity in mice. J Clin Invest. 124:687–695. 24382348 10.1172/JCI67313 PMC3904601

[CIT0009] Dong Z-Y, Zhong W-Z, Zhang X-C, Su J, Xie Z, Liu S-Y, Tu H-Y, Chen H-J, Sun Y-L, Zhou Q. 2017. Potential predictive value of TP53 and KRAS mutation status for response to PD-1 blockade immunotherapy in lung adenocarcinoma. Clin Cancer Res. 23:3012–3024. 28039262 10.1158/1078-0432.CCR-16-2554

[CIT0010] Gao J, Shi LZ, Zhao H, Chen J, Xiong L, He Q, Chen T, Roszik J, Bernatchez C, Woodman SE, et al. 2016. Loss of IFN-γ pathway genes in tumor cells as a mechanism of resistance to anti-CTLA-4 therapy. Cell. Oct. 167:397–404. Epub 2016/09/27. 10.1016/j.cell.2016.08.069 PMC5088716 27667683

[CIT0011] Gerber SA, Sedlacek AL, Cron KR, Murphy SP, Frelinger JG, Lord EM. 2013. IFN-γ mediates the antitumor effects of radiation therapy in a murine colon tumor. Am J Pathol. 182:2345–2354. 23583648 10.1016/j.ajpath.2013.02.041 PMC3668027

[CIT0012] Hyun K-H, Yoon C-H, Kim R-K, Lim E-J, An S, Park M-J, Hyun J-W, Suh Y, Kim M-J, Lee S-J. 2011. Eckol suppresses maintenance of stemness and malignancies in glioma stem-like cells. Toxicol Appl Pharmacol. 254:32–40. 21514314 10.1016/j.taap.2011.04.006

[CIT0013] Kang H-C, Chie EK, Kim HJ, Kim JH, Kim IH, Kim K, Shin BS, Ma E. 2019. A phthalimidoalkanamide derived novel DNMT inhibitor enhanced radiosensitivity of A549 cells by inhibition of homologous recombination of DNA damage. Invest New Drugs. 37:1158–1165. 30793218 10.1007/s10637-019-00730-6

[CIT0014] Kim JH, Kim IH, Shin JH, Kim HJ, Kim IA. 2013. Sequence-dependent radiosensitization of histone deacetylase inhibitors trichostatin A and SK-7041. Cancer Res Treat. 45:334–342. Epub 2014/01/24. 24454006 10.4143/crt.2013.45.4.334 PMC3893331

[CIT0015] Kim JH, Shin JH, Kim IH. 2004. Susceptibility and radiosensitization of human glioblastoma cells to trichostatin A, a histone deacetylase inhibitor. Int J Radiat Oncol Biol Phys. 59:1174–1180. Epub 2004/07/06. 15234053 10.1016/j.ijrobp.2004.03.001

[CIT0016] Lim JY, Gerber SA, Murphy SP, Lord EM. 2014. Type I interferons induced by radiation therapy mediate recruitment and effector function of CD8+ T cells. Cancer Immunol Immunother. 63:259–271. 24357146 10.1007/s00262-013-1506-7 PMC3944132

[CIT0017] Lugade AA, Sorensen EW, Gerber SA, Moran JP, Frelinger JG, Lord EM. 2008. Radiation-induced IFN-γ production within the tumor microenvironment influences antitumor immunity. J Immunol. 180:3132–3139. 18292536 10.4049/jimmunol.180.5.3132

[CIT0018] Ma H, Rao L, Wang H, Mao Z, Lei R, Yang Z, Qing H, Deng Y. 2013. Transcriptome analysis of glioma cells for the dynamic response to γ-irradiation and dual regulation of apoptosis genes: a new insight into radiotherapy for glioblastomas. Cell Death Dis. 4:e895. 24176853 10.1038/cddis.2013.412 PMC3920930

[CIT0019] Mariathasan S, Turley SJ, Nickles D, Castiglioni A, Yuen K, Wang Y, Kadel III EE, Koeppen H, Astarita JL, Cubas R. 2018. TGFβ attenuates tumour response to PD-L1 blockade by contributing to exclusion of T cells. Nature. 554:544. 29443960 10.1038/nature25501 PMC6028240

[CIT0020] McDonald JT, Gao X, Steber C, Lee Breed J, Pollock C, Ma L, Hlatky L. 2017. Host mediated inflammatory influence on glioblastoma multiforme recurrence following high-dose ionizing radiation. PLoS One. 12:e0178155. 28542439 10.1371/journal.pone.0178155 PMC5439715

[CIT0021] McGranahan T, Therkelsen KE, Ahmad S, Nagpal S. 2019. Current state of immunotherapy for treatment of glioblastoma. Curr Treat Options Oncol. 20:24. 30790064 10.1007/s11864-019-0619-4 PMC6394457

[CIT0022] Motzer RJ, Escudier B, McDermott DF, George S, Hammers HJ, Srinivas S, Tykodi SS, Sosman JA, Procopio G, Plimack ER. 2015. Nivolumab versus everolimus in advanced renal-cell carcinoma. N Engl J Med. 373:1803–1813. 26406148 10.1056/NEJMoa1510665 PMC5719487

[CIT0023] Permata TBM, Hagiwara Y, Sato H, Yasuhara T, Oike T, Gondhowiardjo S, Held KD, Nakano T, Shibata A. 2019. Base excision repair regulates PD-L1 expression in cancer cells. Oncogene. 38:4452–4466. Epub 2019/02/14. 30755733 10.1038/s41388-019-0733-6

[CIT0024] Sato H, Niimi A, Yasuhara T, Permata TBM, Hagiwara Y, Isono M, Nuryadi E, Sekine R, Oike T, Kakoti S. 2017a. DNA double-strand break repair pathway regulates PD-L1 expression in cancer cells. Nat Commun. 8:1–11. 29170499 10.1038/s41467-017-01883-9 PMC5701012

[CIT0025] Sato H, Niimi A, Yasuhara T, Permata TBM, Hagiwara Y, Isono M, Nuryadi E, Sekine R, Oike T, Kakoti S, et al. 2017b. DNA double-strand break repair pathway regulates PD-L1 expression in cancer cells. Nat Commun. 8:1751. Epub 2017/11/25. 29170499 10.1038/s41467-017-01883-9 PMC5701012

[CIT0026] Schaue D, McBride WH. 2012. T lymphocytes and normal tissue responses to radiation. Front Oncol. 2:119. 23050243 10.3389/fonc.2012.00119 PMC3445965

[CIT0027] Shao S, Risch E, Burner D, Lu L, Minev B, Ma W. 2017. IFNγ enhances cytotoxic efficiency of the cytotoxic T lymphocytes against human glioma cells. Int Immunopharmacol. 47:159–165. 28410529 10.1016/j.intimp.2017.04.003

[CIT0028] Stupp R, Mason WP, Van Den Bent MJ, Weller M, Fisher B, Taphoorn MJ, Belanger K, Brandes AA, Marosi C, Bogdahn U. 2005. Radiotherapy plus concomitant and adjuvant temozolomide for glioblastoma. N Engl J Med. 352:987–996. 15758009 10.1056/NEJMoa043330

[CIT0029] Trujillo JA, Sweis RF, Bao R, Luke JJ. 2018. T cell-inflamed versus non-T cell-inflamed tumors: a conceptual framework for cancer immunotherapy drug development and combination therapy selection. Cancer Immunol Res. 6:990–1000. 30181337 10.1158/2326-6066.CIR-18-0277 PMC6145135

[CIT0030] Tulip IJ, Kim S-O, Kim E-J, Kim J, Lee JY, Kim H, Kim S-C. 2021. Combined inhibition of STAT and Notch signalling effectively suppresses tumourigenesis by inducing apoptosis and inhibiting proliferation, migration and invasion in glioblastoma cells. Animal Cells Syst (Seoul). 25:161–170. 10.1080/19768354.2021.1942983 PMC8253205 34262659

[CIT0031] Vanpouille-Box C, Diamond JM, Pilones KA, Zavadil J, Babb JS, Formenti SC, Barcellos-Hoff MH, Demaria S. 2015. TGFβ is a master regulator of radiation therapy-induced antitumor immunity. Cancer Res. 75:2232–2242. 25858148 10.1158/0008-5472.CAN-14-3511 PMC4522159

[CIT0032] Vujaskovic Z. 2000. TGF-beta, radiation-induced pulmonary injury and lung cancer. Int J Radiat Biol. 76:511–516. 10815631 10.1080/095530000138510

[CIT0033] Wang Y, Deng W, Li N, Neri S, Sharma A, Jiang W, Lin SH. 2018. Combining immunotherapy and radiotherapy for cancer treatment: current challenges and future directions. Front Pharmacol. 9:185. 29556198 10.3389/fphar.2018.00185 PMC5844965

[CIT0034] Weller M, Le Rhun E. 2019. Immunotherapy for glioblastoma: quo vadis? Nat Rev Clin Oncol. 16:405–406. Epub 2019/03/15. 30867572 10.1038/s41571-019-0195-3

[CIT0035] Wolchok JD, Kluger H, Callahan MK, Postow MA, Rizvi NA, Lesokhin AM, Segal NH, Ariyan CE, Gordon R-A, Reed K. 2013. Nivolumab plus ipilimumab in advanced melanoma. N Engl J Med. 369:122–133. 23724867 10.1056/NEJMoa1302369 PMC5698004

[CIT0036] Wrzesinski SH, Wan YY, Flavell RA. 2007. Transforming growth factor-β and the immune response: implications for anticancer therapy. Clin Cancer Res. 13:5262–5270. 17875754 10.1158/1078-0432.CCR-07-1157

[CIT0037] Zaretsky JM, Garcia-Diaz A, Shin DS, Escuin-Ordinas H, Hugo W, Hu-Lieskovan S, Torrejon DY, Abril-Rodriguez G, Sandoval S, Barthly L. 2016. Mutations associated with acquired resistance to PD-1 blockade in melanoma. N Engl J Med. 375:819–829. 27433843 10.1056/NEJMoa1604958 PMC5007206

