# Supplementary material for: Time-sequential change in immune-related gene expression after irradiation in glioblastoma: next-generation sequencing analysis
Source: Anim Cells Syst (Seoul). 2021 Jul 30;25(4):245–54. doi: 10.1080/19768354.2021.1954550 (PMC8366673; doi:10.1080/19768354.2021.1954550)

Time-sequential change in immune-related gene expression after irradiation in glioblastoma: next-generation sequencing analysis

Supplementary materials

Supplementary Fig S1. Authentication and mycoplasma tests of the U373 MG and A549 cell lines.

(a) Authentication test in the U373 MG and A549 cell lines

- U373 MG

Locus	Reference Database Profile<br>Database : U-373MG (CVCL_2219)		Sample Profile<br>Sample Name : U373MG		Shared alleles #
D5S818	11	12	11	12	2
D13S317	10	11	10	11	2
D7S820	10	12	10	12	2
D16S539	12		12		1
vWA	16	18	16	18	2
TH01	9.3		9.3		1
TPOX	8		8		1
CSF1PO	11	12	11	12	2
AMEL	X	Y	X	Y	2
D3S1358	16	17	16	17	2
D21S11	29	30	29	30	2
D18S51	13		13		1
D8S1179	13	15	13	15	2
FGA	21	25	21	25	2
D2S1338			24		-
D19S433			13	15	-
Penta D	10	12	10	12	2
Penta E	7	10	7	10	2
Number of shared alleles					28
Total number of alleles in the reference database profile					28
% match					100.0%
**Result interpretation**					**Related**

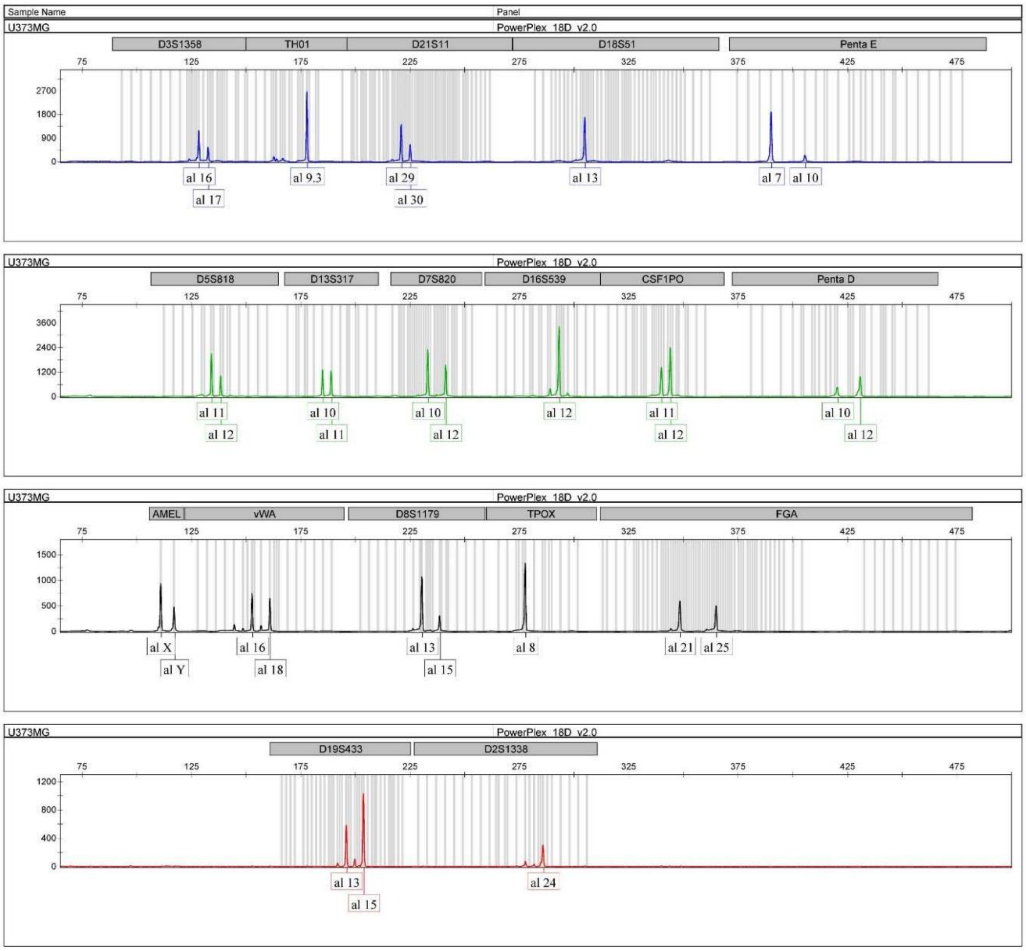

- A549

Locus	Reference Database Profile		Sample Profile		Shared alleles #
	Database : A549 (ATCC CCL-185)		Sample Name : A549		
D5S818	11		11		1
D13S317	11		11		1
D7S820	8	11	8	11	2
D16S539	11	12	11	12	2
vWA	14		14		1
TH01	8	9.3	8	9.3	2
TPOX	8	11	8	11	2
CSF1PO	10	12	10	12	2
AMEL	X	Y	X	Y	2
D3S1358			16		-
D21S11			29		-
D18S51			14	17	-
D8S1179			13	14	-
FGA			23		-
D2S1338			24		-
D19S433			13		-
Penta D			9		-
Penta E			7	11	-
Number of shared alleles					15
Total number of alleles in the reference database profile					15
% match					100.0%
**Result interpretation**					**Related**

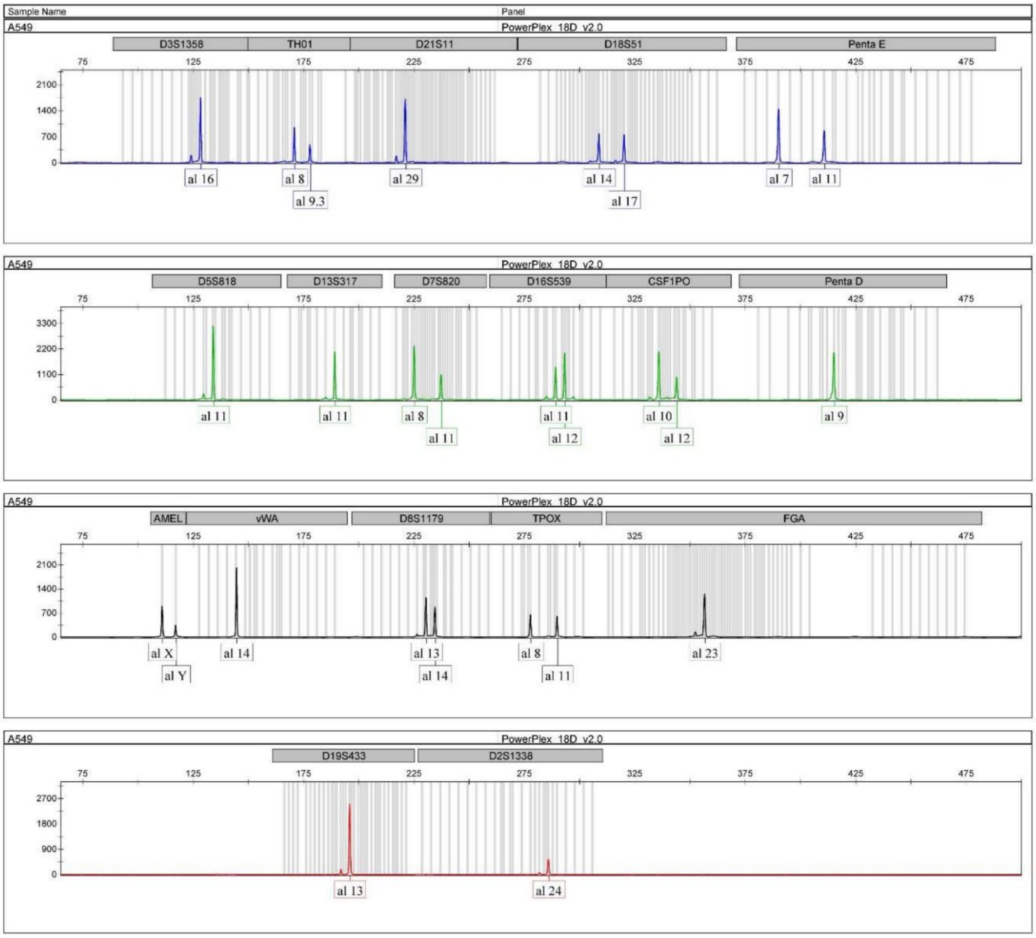

(b) Mycoplasma test in the U373 MG and A549 cell lines

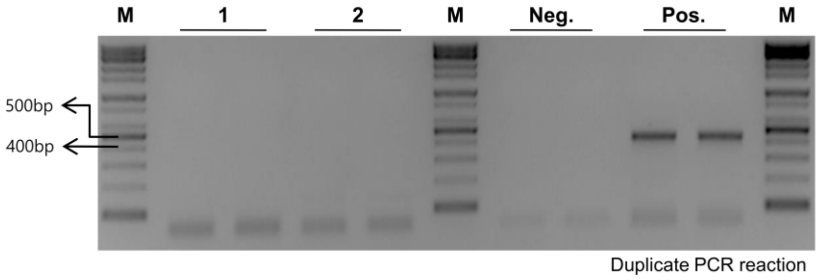

No.	Sample name	Cell Line	PCR results	Sequencing & BLAST
1	U373MG	U373MG	-	
2	A549	A549	-	

Abbreviations: M, mycoplasma; Neg., negative; Pos., positive.

Supplementary Fig S2. Repeated western blotting for each time period post-irradiation of the U373 MG glioblastoma cell line (Set#2). IR, irradiation; Cont, control.

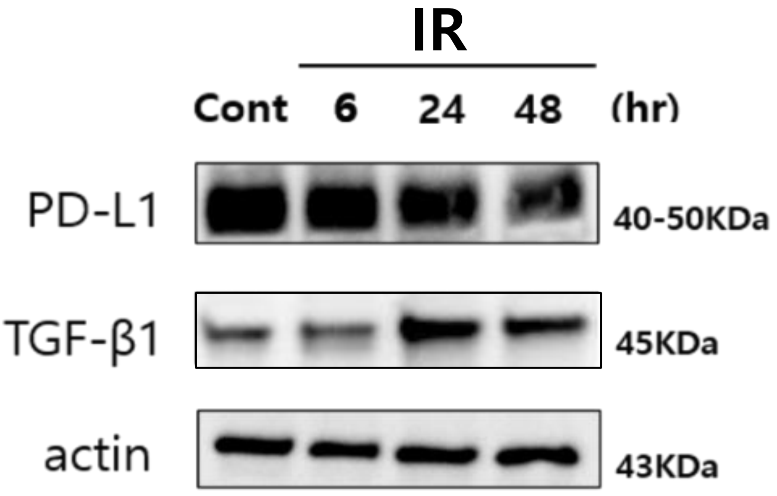

Supplementary Fig S2-2. Repeated western blotting of TGF-β1 for each time period post-irradiation of the U373 MG glioblastoma cell line (Set#3). IR, irradiation; Cont, control.

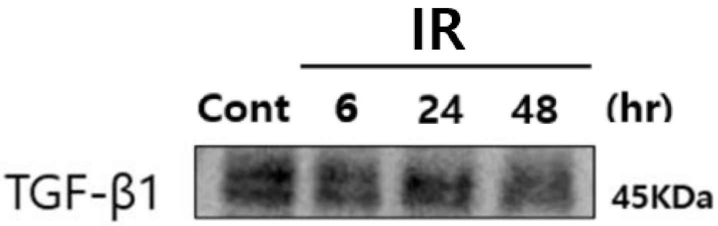

Supplementary Fig S3. Full-size blots of Fig 3 (a, Set#1) and supplementary Fig S2 (b, Set#2). IR, irradiation; Cont, control.

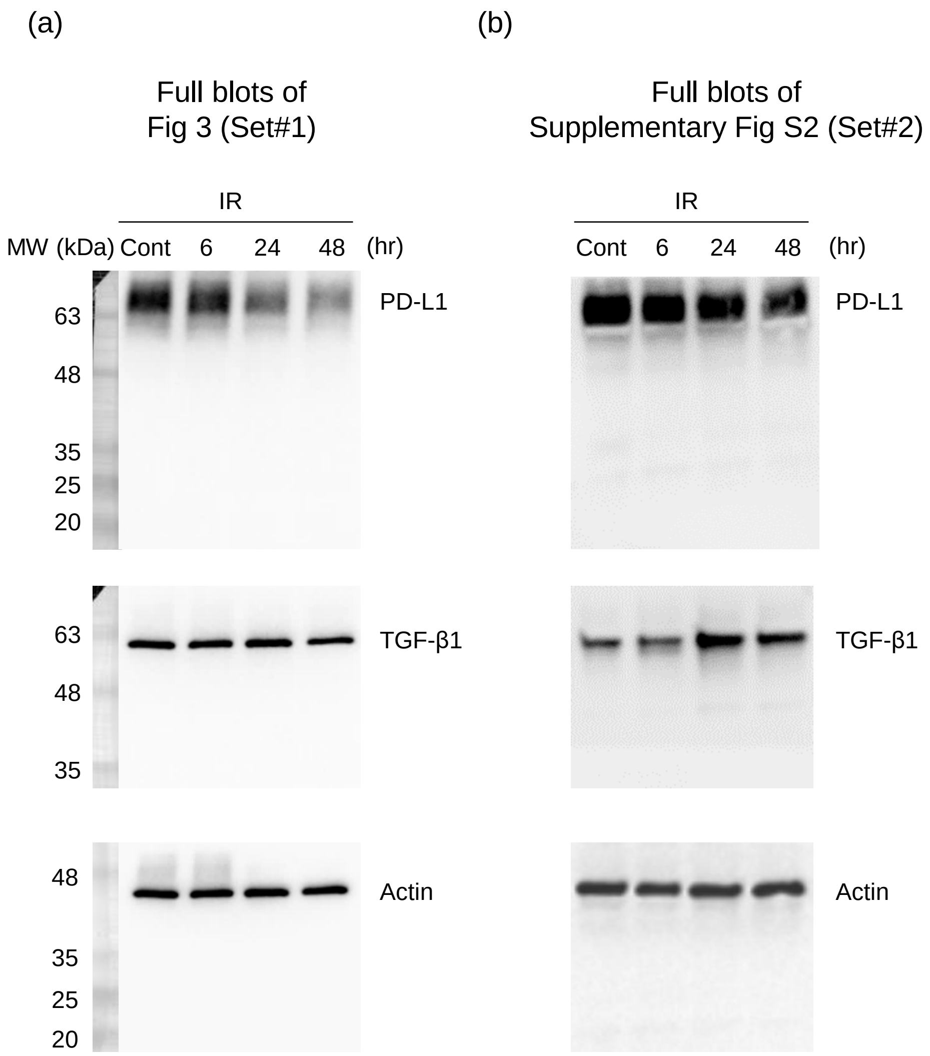

Supplement: Supplemental Material [file TACS_A_1954550_SM4920.zip › supplementary_figures.pdf]
